# Effects of phenobarbitone on neonatal hyperbilirubinemia, a systematic review and meta-analysis of randomized controlled trial

**DOI:** 10.1186/s12887-025-05844-w

**Published:** 2025-07-02

**Authors:** Jie Zhang, Bo Zheng, Jianbo Zhuang, Yu Chen, Xiaming Xu, Xinfa Ren

**Affiliations:** 1 Suzhou Ninth hospital affiliated to Soochow University, Soochow, China; 2 UNC of Greensboro, Greensboro, USA; 3 Yuhuan Second People’s Hospital, Zhejiang, China

**Keywords:** Phenobarbitone, Phototherapy, RCTs (randomized controlled trials), Neonatal hyperbilirubinemia, Meta-analysis

## Abstract

**Background:**

The management of neonatal jaundice is relatively straightforward; however, hemodynamic insults may still occur, particularly in very low birth weight infants. While phototherapy is effective, it may lead to prolonged hospitalization and potential adverse effects. This study investigates the efficacy of phenobarbitone in preventing neonatal hyperbilirubinemia in both term and preterm neonates.

**Method:**

We conducted a comprehensive search of PubMed, Web of Science, Embase, Cochrane Library, Scopus, and CINAHL to identify relevant articles published up to January 23, 2025. Study selection followed the PRISMA guidelines, and assessments of the risk of bias were performed. Data analysis was conducted using Review Manager 5.3, with mean difference (MD) and 95% confidence intervals (CI) calculated for continuous variables, while risk ratios (RR) were estimated for binary variables.

We included randomized controlled trials (RCTs) that investigated the effects of phenobarbitone on neonatal hyperbilirubinemia. The primary outcomes assessed were phototherapy duration, the need for exchange transfusion, peak bilirubin levels, the need of phototherapy, and cumulative weight loss. The extracted data encompassed key study characteristics along with an evaluation of the risk of bias 1.0 tool and Level of Evidence was summarized using GRADE guidelines.

**Result:**

A total of 41 articles were identified through searches of six databases. After removing duplicates, 23 articles remained. Based on title and abstract screening, 15 were excluded. An additional 3 articles were excluded for not meeting the inclusion criteria. Five studies, including 590 newborns, were identified and included in the meta-analysis. This meta-analysis discovered significant changes in phototherapy duration, measured in hours (mean difference (MD) –24.51 (95% CI –34.08, –14.94), *p* < 0.01) with low heterogeneity, exchange transfusion(Risk ratio(RR) 0.32 (95% CI 0.16, 0.61), *p* < 0.01) with low heterogeneity, peak bilirubin level(MD –2.35 (95% CI –2.96, –1.74), *p* < 0.01) with low heterogeneity, need of phototherapy (RR 0.75 (95% CI 0.66, 0.85), *p* < 0.01) with low heterogeneity. There was not a significant change in weight loss (MD 0.15 (95% CI -0.76, 1.07), *p* = 0.74) with low heterogeneity. Only one of these studies reported side effects of phenobarbitone (Pediatric Res 51:399A, 2002).

**Conclusion:**

Phenobarbitone might be applied in unconjugated neonatal hyperbilirubinemia to reduce the peek bilirubin level, phototherapy duration, and reduce the chance of exchange transfusion and phototherapy treatment.

**PROSPERO registration number:**

CRD42025636128.

**Supplementary Information:**

The online version contains supplementary material available at 10.1186/s12887-025-05844-w.

## Introduction

Neonatal jaundice is a significant global health concern [1]. Neonatal jaundice is a significant concern due to its potential association with neurotoxicity, making it a source of anxiety for both parents and physicians. If left untreated or inadequately managed, it can result in neonatal mortality or severe complications, including seizures in the temporal and occipital lobes, commonly known as bilirubin encephalopathy [2].

While phototherapy remains a primary treatment, exchange transfusion is employed in cases of severe jaundice. However, exchange transfusion carries substantial risks, including morbidity and mortality, as well as complications associated with blood product exposure. The risk is further exacerbated in small and preterm infants [3]. Therefore, a prophylactic intervention capable of preventing total serum bilirubin (TSB) from reaching dangerous levels would be a more effective approach for both term and preterm neonates.

Among these approaches, phototherapy has become the most widely used non-pharmacological method for preventing and treating unconjugated neonatal hyperbilirubinemia. Its widespread adoption is attributed to its simplicity, non-invasiveness, cost-effectiveness, and ease of application [4]. Phototherapy converts bilirubin into a water-soluble form that is easily eliminated, leading to a reduction in total serum bilirubin (TSB) levels. Light in the blue-green spectrum, with wavelengths ranging from 425 to 475 nm, interacts biochemically with bilirubin deposited in the skin of jaundiced neonates, promoting its breakdown and excretion [5].

Phototherapy units are widely used to treat unconjugated neonatal hyperbilirubinemia, they are challenging and costly to maintain. However, it might induce hyperthermia, skin rash, diarrhea, dehydration, and retinal damage and does not prevent the accumulation of bilirubin and the need for ET in all cases [6]. As an alternative for the prevention and treatment of jaundice, phenobarbitone has been shown to be both safe and effective in lowering serum bilirubin levels in neonates [7, 8]. Phenobarbitone reduces jaundice by promoting the excretion of bilirubin. It enhances glucuronidation through the induction of hepatic microsomal enzymes and increases the production of receptor proteins for bilirubin uptake [9]. However, Phenobarbitone also has some side effects, such as hypotension and respiratory depression, and it can lead to cognitive decline in infants [10]. We will also provide an analysis of the potential adverse effects of phenobarbital.

Both phenobarbitone and phototherapy are effective in reducing the peak serum bilirubin (PSB) level.

We aimed to conduct a systematic review and meta-analysis to evaluate the impact of phenobarbitone on reducing the need for phototherapy, the duration of phototherapy, peak bilirubin levels, the likelihood of exchange transfusion, and weight loss.

## Methods

### Eligibility criteria

The PRISMA guidelines were followed as a checklist for this study, and the study protocol was registered on PROSPERO. The link of PROSEPRO: https://www.crd.york.ac.uk/PROSPERO/view/CRD42025636128. The inclusion criteria were based on the PICOS framework, as follows: the study population consisted of newborns with unconjugated hyperbilirubinemia, which was obtained by TSB (total serum bilirubin). The intervention group received phenobarbitone, both saturated and unsaturated doses, as well as both oral and intravenous administrations, were included in the study. While the control group received either placebo with phototherapy or phototherapy alone. The primary outcome of the study was duration of phototherapy, peak bilirubin level, likelihood of exchange transfusion. The secondary outcome was the weight loss which measured in gram, and the number requiring phototherapy. Only randomized controlled trials with accessible full-text publications were included, with no language restrictions. The exclusion criteria were as follows: (1) observational studies with control (cohort or case–control) or without control (cross-sectional studies or case series); (2) unpublished abstracts; and (3) non-primary research.

### Search strategy

Literature was systematically searched in six databases (EMBASE, PubMed, Cochrane Library, Web of Science, Scopus, and CINAHL). The data was updated up to January 23, 2025. The following keywords were utilized in the search: “phenobarbitone”,” jaundice”, “hyperbilirubinemia”, “neonatal”,” newborn”, “neonate”, and “random”. The use of operators (AND, OR) and adjustments for the specific search criteria of each database were done. A manual search was also conducted on each of the reference lists of the included studies. The search strategy and the respective databases can be found in the supplementary materials.

### Study selection

Two researchers independently screened the study titles and abstracts. They evaluated the selected articles by full-text review and assessed their eligibility. Any disagreements were resolved by consulting with the third member of the review team.

### Data extraction

The study details included the first author, year of publication, sample size, study population, phenobarbitone dosage, the basic information (like gestational age, weight, sex), and the outcome. All data were retrieved by two researchers. Discrepancies were settled by discussion or, if necessary, consultation with the third member of the review team. When details were not accessible in published publications, we contacted authors for further information or clarifications.

### Study risk of bias assessment

Two authors used Review Manager 5.4 [11] to assess the risk of bias from all studies, such as Random Sequence Generation, Allocation Concealment, Blinding, Incomplete Outcome Data, Selective Reporting, and Other Sources of Bias. The assessment results are classified into high, low, or unclear.

### Data synthesis

The Review Manager 5.4 (Cochrane Collaboration) software and StataMP 14.0 were used for this meta-analysis. The Inverse Variance method was used to calculate the mean difference (MD) and 95% confidence interval (CI) for continuous variables. A random-effects model was applied if heterogeneity was high; otherwise, a fixed-effects model was used. The risk ratio (RR) for binary variables. Heterogenicity was measured with I^2^. We defined heterogeneity as low, moderate, and high degrees when I^2^ < 25%, 26–50%, and > 50% [12]. Results were considered significant if the two-tailed *p*-value was ≤ 0.05. Due to the limited availability of the included original data, we did not take dosage into consideration. Therefore, it is necessary to merge the data from the two articles [13, 14]. We used the following formula to merge continuous variables.

$${\text{mean}}_{\text{combined}}=\frac{n1\cdot {\text{mean}}_{1} +{n}_{2} \cdot {\text{mean}}_{2}}{{n}_{1} +{n}_{2}}$$

$${\text{SD}}_{\text{combined}}= \sqrt{\frac{{n}_{1}\cdot \left({\text{SD}}_{1}^{2} + {\left({\text{mean}}_{1} -{\text{mean}}_{\text{combined}}\right)}^{2}\right) +{n}_{2} \cdot \left({\text{SD}}_{2}^{2} +{\left({\text{mean}}_{2} -{\text{mean}}_{\text{combined}}\right)}^{2}\right)}{{n}_{1} +{n}_{2}}}$$

### Assessment of publication bias

This is Examination of publication bias will not be generated in this study as a funnel plot analysis typically requires a minimum of 10 studies to ensure sufficient power to detect heterogeneity among studies. However, it is essential to acknowledge that the study may be susceptible to publication bias due to the limited number of available publications [11].

### GRADE

Grading of Recommendations Assessments, Development and Evaluation (GRADE) analysis was applied to the outcomes to evaluate the quality of evidence and was completed [15].

## Results

### Studies selection

**Fig. 1:**
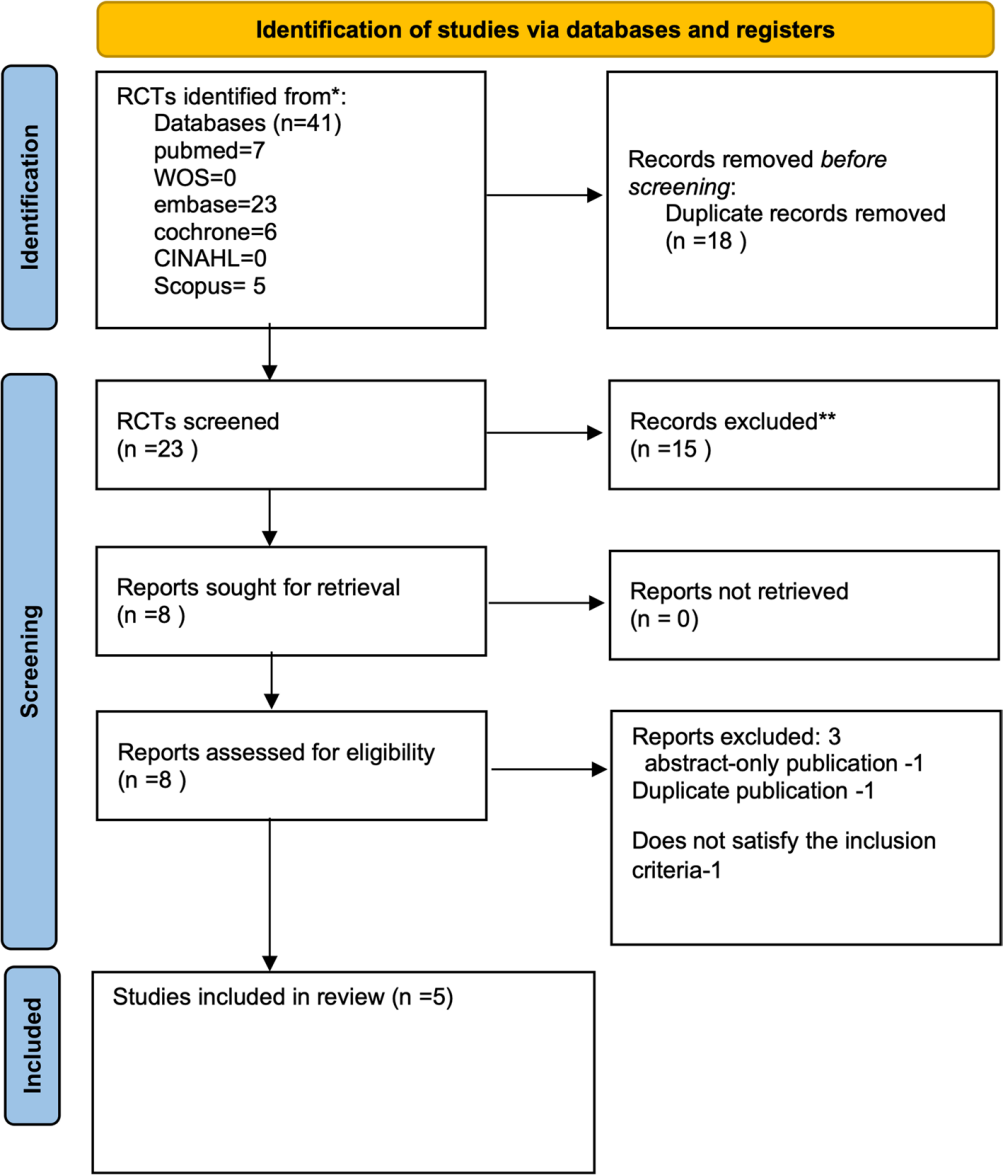
The PRISMA flow diagram. PRISMA, preferred reporting items for systematic reviews and meta-analyses

A total of 41 articles were retrieved in the initial search from the databases. Among them, eighteen articles were removed as duplicates. eighteen studies were then excluded out of the retrieved articles. Ultimately, five records were included in this systematic review. A detailed PRISMA flow diagram for the study selection process is shown in Fig. 1.

### Characteristics of included studies

One study was correspondence [16] and others were articles. One study was conducted in Finland [17], other four studies in India [13, 14, 16, 18]. The minimum and maximum number of participants was 75 [18] and 164 [14]. Three studies involved preterm neonates [13, 14, 17], two studies included term neonates in their study [16, 18]. The control group in one studies received placebo (glucose) in the remaining studies [18], four studies received no intervention in the remaining studies. three the intervention groups received phenobarbitone by Intravenous Infusion [13, 14, 17], two intervention groups received phenobarbitone by oral [16, 18]. The dosage and timing of medication administration varied across studies; for details, refer to Table 1. The extracted data and included studies’ characteristics are shown in Table 1. Since this study is a meta-analysis, so the table only presents research findings included in at least two studies. For example, study on drug side effects was not included, as only one study [18] provided relevant data.

**Table 1:** Main characteristics of included studies for the effect of phenobarbitone compared to control

study ID	Year	Country	Group	N	Gestational Age (weeks)	Birth Weight (g)	Outcome	Peak Bilirubin (mg/dl)	Phototherapy Case	Phototherapy Duration (h)	Exchange Transfusion (n)	Weight Loss at 72 h (g)	Phenobarbital Usage	Male (%)	Feeding Method	Randomization Method
Vineta Ruth	1988	Finland	Phenobarbital	47	29.4 ± 1.7	1160 ± 210	Peak bilirubin	6.8 ± 1.8	-	-	-	-	IV: 5 mg/kg once, then 15 mg/kg after 4 h, then 5 mg/kg/day × 5d	44.6	unclear	Lottery
			Control	54	29.2 ± 2.1	1120 ± 250	Peak bilirubin	9.2 ± 2.1	-	-	-	-	IV glucose infusion	50		
R. Aggarwal^a^	2001	India	Phenobarbital	50	39.3 ± 1.0	2821 ± 360	Multiple	-	14	44 ± 28	1	4.0 ± 3.4	Oral 3 mg/kg/day × 5d	Unclear	Exclusive BF	unclear
			Control	50	39.3 ± 0.9	2910 ± 373	Multiple	-	26	66 ± 45	3	4.5 ± 3.7	NA	Unclear		
Arya	2002	India	Phenobarbital	37	38.8 ± 1.1	2930 ± 288	Multiple	-	2	-	-	5.5 ± 2.49	Oral 5 mg/kg/day × 3d	54.1	Exclusive BF	Computer generated
			Placebo	38	38.7 ± 1.2	2841 ± 258	Multiple	-	4	-	-	4.85 ± 2.86	Oral glucose in milk	60.5		
Kumar	2002	India	Phenobarbital	50	31.36 ± 2.78	1257 ± 142	Multiple	10.14 ± 2.59	35	85.6 ± 38.4	4	-	IV: 10 mg/kg dose on day 1, 5 mg/kg/day from day 2 to day 5	71.98	unclear	Computer generated
			Phenobarbital	50	31.84 ± 2.43	1268 ± 157	Multiple	10.16 ± 3.15	32	113.7 ± 54.9	7	-	IV: dose of 5 mg/kg/day from day 1 to day 5	45.94		
			Control	50	31.2 ± 2.4	1257 ± 246	Multiple	12.17 ± 3.36	43	115 ± 62.7	16	-	NA	51.9		
Hussain	2022	India	Phenobarbital	64	Unclear	1000–1499	Multiple	8.62 ± 3.46	41	82.05 ± 36.61	-	-	IV:5 mg/kg per day for 5 days starting within 6 h of birth	unclear	unclear	unclear
			Phenobarbital	54	Unclear	1000–1499	Multiple	10.10 ± 3.25	40	120.26 ± 39.86	-	-	IV:5 mg/kg per day for 5 days starting after 2 days of birth	unclear		
			Control	50	Unclear	1000–1499	Multiple	13.45 ± 10.95	43	132.81 ± 49.7	-	-	NA	unclear		

*NA* not available, *IV* Intravenous, *BF* breastmilk feeding

^a^Correspondence

### Risk of biases assessment

**Fig. 2:**
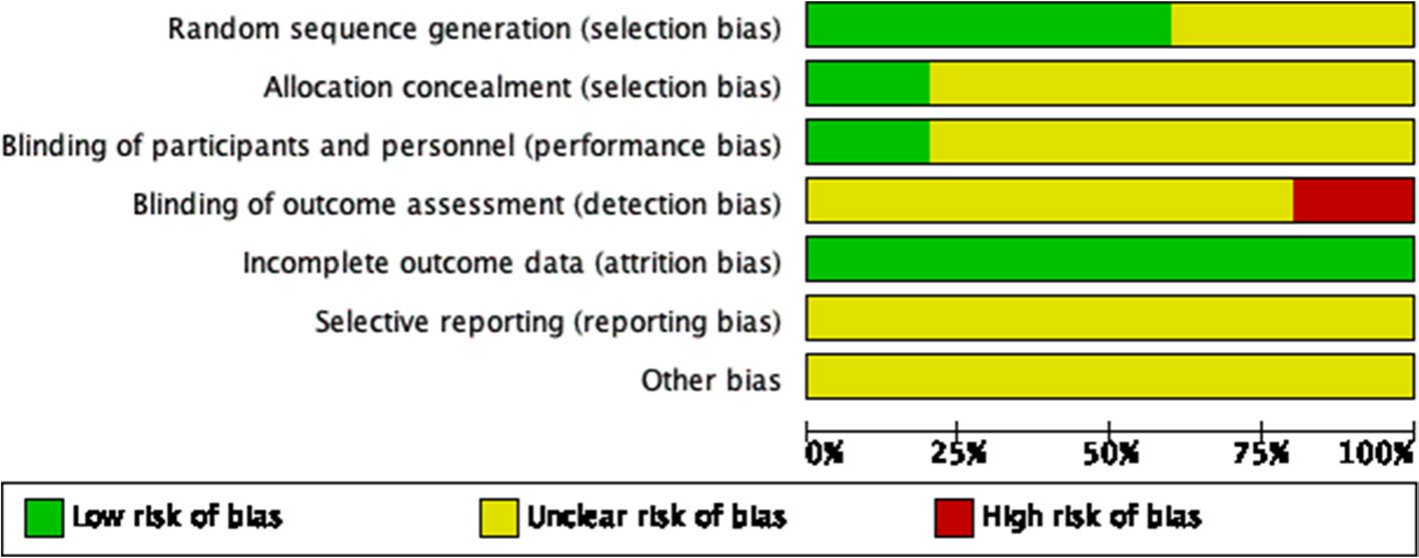
Graph for the risk of bias

**Fig. 3:**
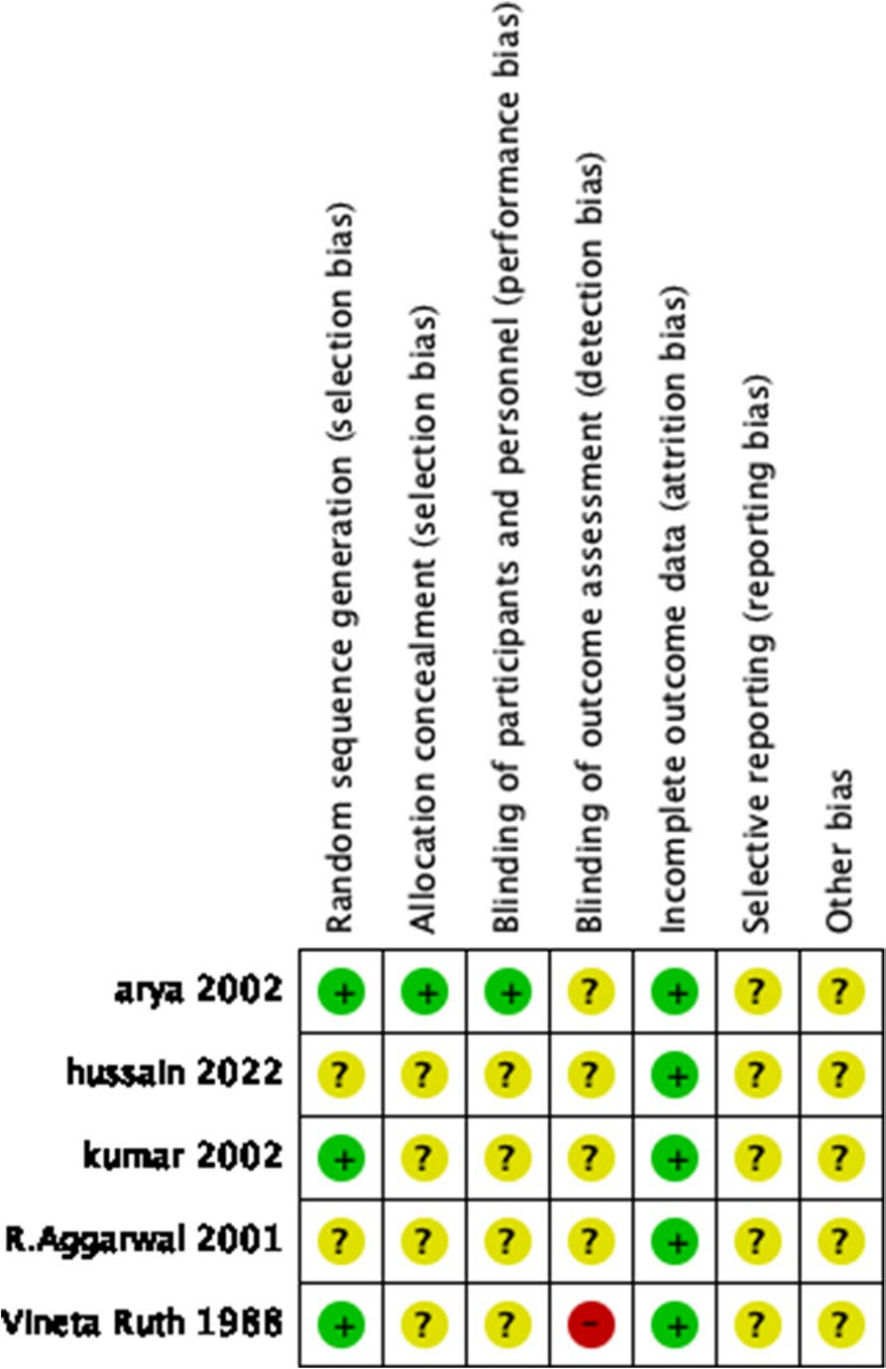
Summary of the risk of bias

The visual representation of bias assessment is shown as a risk of bias graph (Fig. 2) and table (Fig. 3). The assessment showed moderate results of bias, except for one study, which showed a high result of bias in blinding of outcome assessment [17]. All studies used randomization, but details of the randomization method were unclear in two studies [14, 16].

### Primary outcomes

#### Peak bilirubin level (mg/dL)

**Fig. 4:**
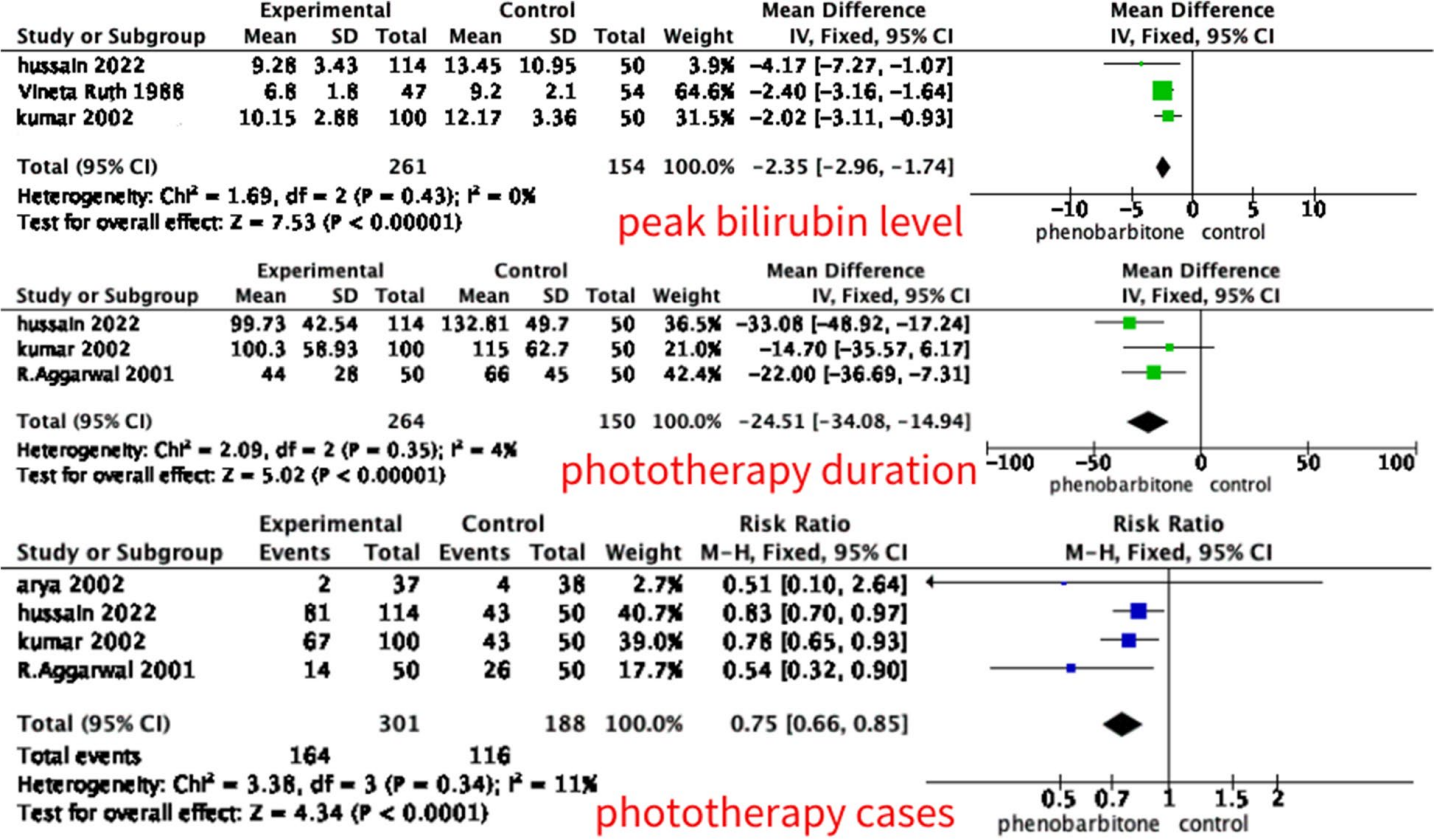
Forest plot for the effect of phenobarbitone

Three included studies (*n* = 451) reported peak bilirubin level within the first 7 days of life. The pooled analysis showed that a significant decrease in peak bilirubin level compared to control was associated with the phenobarbitone and control combination group (MD –2.35 (95% CI –2.96, –1.74), *p* < 0.01, fixed effect model), with a low degree heterogeneity (I^2^ = 0%), the detail is shown in Fig. 4. Due to the limited number of included studies and the low heterogeneity, sensitivity analysis and subgroup analysis were not conducted.

#### Phototherapy duration

Three included studies (*n* = 414) reported phototherapy duration, the intensity of phototherapy was not reported. The pooled analysis showed that a significant decrease in phototherapy duration compared to control was associated with the phenobarbitone and control combination group (MD –24.51 (95% CI –34.08, –14.94), *p* < 0.01, fixed effect model), with a low degree heterogeneity (I^2^ = 4%), the detail is shown in Fig. 4. Due to the limited number of included studies and the low heterogeneity, sensitivity analysis and subgroup analysis were not conducted.

#### Exchange transfusion

Two included studies (*n* = 250) reported exchange transfusion. The pooled analysis showed that a significant decrease in exchange transfusion compared to control was associated with the phenobarbitone and control combination group (RR 0.32 (95% CI 0.16, 0.61), *p* < 0.01, fixed effect model), with a low degree heterogeneity (I^2^ = 0%), the detail is shown in Fig. 4.

### Secondary outcomes

#### Weight loss(g)

**Fig. 5:**
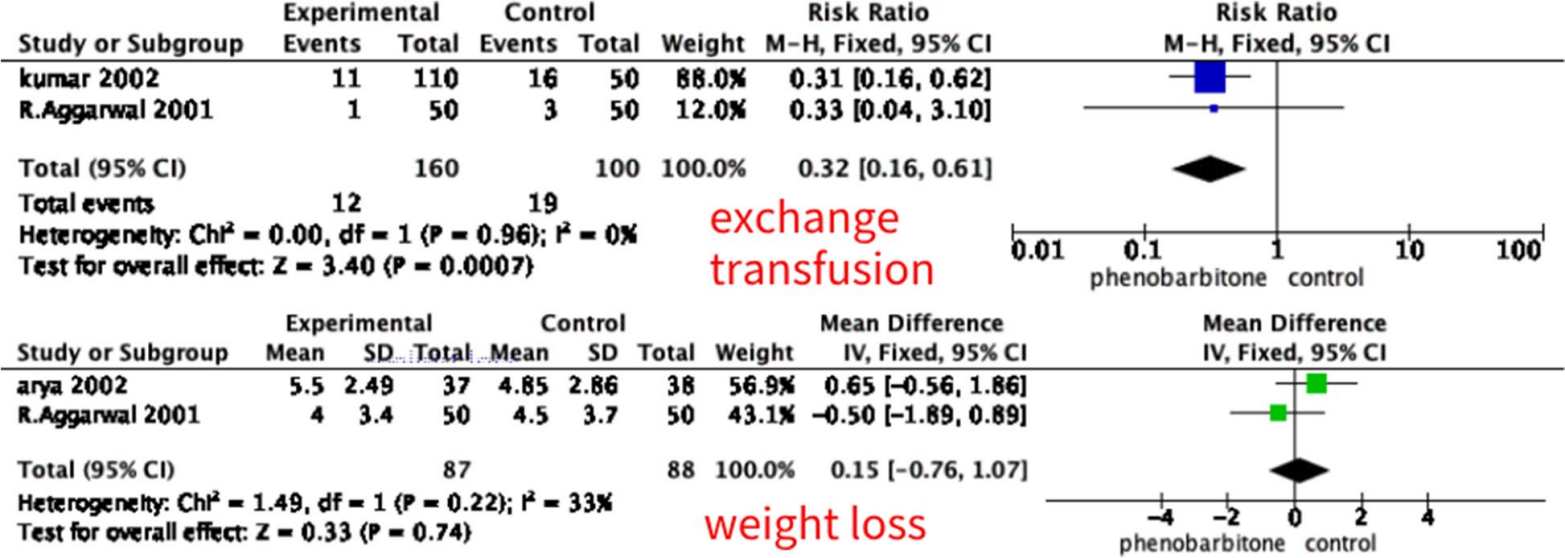
Forest plot for the effect of phenobarbitone for exchange transfusion and weight loss

Two included studies (*n* = 175) reported weight loss measured in gram after intervention. The pooled analysis showed that the weight loss was insignificant compared to the control group associated with the phenobarbitone and control combination group (MD 0.15 (95% CI –0.76, 1.07), *p* = 0.75, fixed effect model), with a low degree heterogeneity (I^2^ = 33%), the detail is shown in Fig. 5.

#### Phototherapy cases

Four included studies (*n* = 489) reported phototherapy number. The pooled analysis showed that a significant decrease in phototherapy number compared to control was associated with the phenobarbitone and control combination group (RR 0.75 (95% CI 0.66, 0.85), *p* < 0.01, fixed effect model), with a low degree heterogeneity (I^2^ = 11%), the detail is shown in Fig. 5. Due to the limited number of included studies and the low heterogeneity, sensitivity analysis and subgroup analysis were not conducted.

### The GRADE of these study

A GRADE assessment of the meta-analyses outcomes for objective measures of urinary incontinence showed that there is a low certainty of evidence for these results, which are shown in the Table 2. Four outcomes were downgraded due to inadequate randomization methods, one outcome was downgraded for imprecision due to wide confidence intervals, and all outcomes were downgraded for potential publication bias as the limited number of included studies made it difficult to rule out its presence.

**Table 2:** The GRADE of outcomes

	**study limitation**	**imprecision**	**inconsistency**	**indirectness**	**publication bias**	**GRADE**
Peak Bilirubin	Downgraded due to unclear randomization methods in some studies	no downgrade	no downgrade	no downgrade	Downgraded due to the limited number of included studies	low
Phototherapy Case	Downgraded due to unclear randomization methods in some studies	no downgrade	no downgrade	no downgrade	Downgraded due to the limited number of included studies	low
Phototherapy Duration	Downgraded due to unclear randomization methods in some studies	no downgrade	no downgrade	no downgrade	Downgraded due to the limited number of included studies	low
Exchange Transfusion	Downgraded due to unclear randomization methods in some studies	no downgrade	no downgrade	no downgrade	Downgraded due to the limited number of included studies	low
Weight Loss at 72 h	no downgrade	Downgraded due to wide confidence intervals	no downgrade	no downgrade	Downgraded due to the limited number of included studies	low

## Discussion

We conducted this meta-analysis to evaluate the efficacy of phenobarbitone in managing hyperbilirubinemia in both preterm and term neonates. Five studies met the criteria for inclusion in the meta-analysis. A significant reduction was observed in peak serum bilirubin levels, duration of phototherapy, the need for phototherapy, and the likelihood of exchange transfusion with phenobarbitone use, although no significant effect was found on weight loss.

Phenobarbitone reduces the occurrence of jaundice primarily by enhancing hepatic enzyme activity, especially by inducing the expression of UDP-glucuronosyltransferase (UGT). This enzyme plays a critical role in the conjugation and subsequent excretion of bilirubin. By accelerating bilirubin metabolism, phenobarbitone facilitates its conversion into a water-soluble form, promoting elimination through bile and reducing its accumulation in the bloodstream. Furthermore, phenobarbitone enhances hepatic uptake and clearance of unconjugated bilirubin, further lowering serum bilirubin levels. These mechanisms contribute to its effectiveness in preventing and managing jaundice, particularly in neonates at risk of hyperbilirubinemia.

As there is only one study investigating the adverse effects, making it impossible to conduct a meta-analysis. However, Phenobarbital requires a loading dose of 10 mg/kg/day in two divided doses 1 to 2 h apart to achieve equilibration in the various body compartments. In addition, phenobarbital has a very long half-life (t1/2), and it may be necessary to monitor serum levels [19].

One of the five included studies have used loading dose of phenobarbitone [17]. Two intervention groups were used, with one group receiving a loading dose of 20 mg/kg. The study reported that the beneficial effects of phenobarbitone were more pronounced when the loading dose was administered at the start of phototherapy. Pharmacokinetic evaluation of phenobarbitone administered without a loading dose showed an increase in plasma drug levels throughout the treatment period, with no achievement of a steady state even after 7 days of therapy [12]. Therefore, the administration of a loading dose and its appropriate amount may be crucial for achieving the clinical benefits of phenobarbitone. The collected data did not include studies on side effects, as only one study analyzed adverse effects, preventing the possibility of a meta-analysis on this topic. This highlights the need for further research to conduct a comprehensive safety assessment of phenobarbitone therapy.

We also acknowledge several limitations in our study. First, the included studies did not provide detailed descriptions or standardized criteria for the timing of phototherapy. Although no significant heterogeneity was observed, this limitation still reduced the credibility of the meta-analysis. Second, based on the Cochrane risk of bias assessment, while most of the included studies described the random allocation method, some did not provide details on allocation concealment and blinding of personnel. This made it difficult to determine whether proper participant and personnel blinding were implemented. Third, the included studies were limited to short observation periods and did not include long-term follow-up. The methodological quality of clinical trials evaluating phenobarbitone for neonatal jaundice needs further improvement. Fourth, the study may be susceptible to publication bias due to the limited number of available publications. Therefore, well-designed, double-blind, placebo-controlled trials with larger sample sizes are necessary to more definitively determine the efficacy of phenobarbitone.

## Conclusion

This systematic review and meta-analysis demonstrated that beneficial effect of phenobarbitone in reducing hyperbilirubinemia, need of treatment and treatment related morbidities may be of special relevance to a resource-restricted setting where availability of working phototherapy units and adequately trained manpower to perform exchange transfusion is limited. However, due to the quality of the included studies and the limitations of the sample sizes, the long-term efficacy of this therapy requires further confirmation through high-quality, long-term research.

## Supplementary Information

Supplementary Material 1.

## Data Availability

All data used in this meta-analysis were extracted from previously published studies, which are cited in the manuscript. No new data were generated.

## Abbreviations

RCTs: Randomized controlled trials
MD: Mean difference
CI: Confidence interval
RR: Risk ratio
