# Supplementary material for: Effects of phenobarbitone on neonatal hyperbilirubinemia, a systematic review and meta-analysis of randomized controlled trial
Source: BMC Pediatr. 2025 Jul 2;25:504. doi: 10.1186/s12887-025-05844-w (PMC12219915; doi:10.1186/s12887-025-05844-w)
Supplement: Supplementary file 1 — Supplementary Material 1. [file 12887_2025_5844_MOESM1_ESM.pdf]

Search num	Query	Sort By	Filters	Search Det	Results	Time
5	(((Phenobar	Most Recent		"Phenobarb	7	21:48:48
4	random	Most Recent		"random al	1,611,807	21:48:38
3	((((neonatal	Most Recent		"neonatal"[	478,869	21:45:25
2	(jaundice[T	Most Recent		"jaundice"[	47,574	21:44:16
1	Phenobarbi	Most Recent		"Phenobarb	2,315	21:43:18
